# Can free open access resources strengthen knowledge-based emerging public health priorities, policies and programs in Africa?

**DOI:** 10.12688/f1000research.8662.1

**Published:** 2016-05-09

**Authors:** Ernest Tambo, Ghislaine Madjou, Christopher Khayeka-Wandabwa, Emmanuel N. Tekwu, Oluwasogo A. Olalubi, Nicolas Midzi, Louis Bengyella, Ahmed A. Adedeji, Jeanne Y. Ngogang

**Affiliations:** 1 Department of Biochemistry and Pharmaceutical Sciences, Universite des Montagnes, Bangangté, Cameroon; 2 Africa Disease Intelligence and Surveillance, Communication and Response (Africa DISCoR) Foundation, Yaoundé, Cameroon; 3 Africa Population and Health Research Center (APHRC), Nairobi, Kenya; 4 Noguchi Memorial Institute for Medical Research (NMIMR), College of Health Science, University of Ghana, Greater Accra Region, Ghana; 5 Department of Public Health, Kwara State University (KWASU), Malete, Kwara State, Nigeria; 6 National Institute of Health Research, Harare, Zimbabwe; 7 Department of Biomedical Sciences, School of Basic and Biomedical Sciences, University of Health and Allied Sciences (UHAS), Ho, Volta Region, Ghana; 8 Department of Pharmacology and Therapeutics, Kampala International University, Kansaga, Kampala, Uganda; 9 Service de Biochimie, Centre Hospitalier Universitaire (CHU), Yaoundé, Cameroon

**Keywords:** Open access, free access, information, policy, evidence, program, intervention, Africa

## Abstract

Tackling emerging epidemics and infectious diseases burden in Africa requires increasing unrestricted open access and free use or reuse of regional and global policies reforms as well as timely communication capabilities and strategies. Promoting, scaling up data and information sharing between African researchers and international partners are of vital importance in accelerating open access at no cost. Free Open Access (FOA) health data and information acceptability, uptake tactics and sustainable mechanisms are urgently needed. These are critical in establishing real time and effective knowledge or evidence-based translation, proven and validated approaches, strategies and tools to strengthen and revamp health systems.  As such, early and timely access to needed emerging public health information is meant to be instrumental and valuable for policy-makers, implementers, care providers, researchers, health-related institutions and stakeholders including populations when guiding health financing, and planning contextual programs.

## Introduction

In recent times, the persistence and unprecedented emergence of rising epidemics and infectious diseases in Africa and worldwide triggered numerous public health declarations of international concern ^1^. These local and global uncertainties and potential consequences have prompted questions and reflections on the usefulness and implications of unlocked and unprocessed available massive database resources from different national, regional and international funded projects overtime in low and middle income countries (LMICs) and particularly in Africa. Can free open access (FOA) to these valuable resources improve evidence-based decision making policies, health planning and adequate funding allocation, innovative programs and strategic interventions performance and effectiveness to most vulnerable populations’ health and socio-economic benefits? Emerging and re-emerging infectious diseases epidemics are rampant, ranging from Ebola, influenza, Lassa fever, HIV SARS and MERS-CoV, Zika to other zoonotic diseases existing as potential threats with sporadic epidemics in old and new regions ^2, 3^.

There is a need for availability of data and information on human-vector-pathogen-ecosystem interfaces, drugs and vaccines development as well as diagnostics techniques and tools from preclinical to clinical levels. It is critical that the information is used in an equitable, ethical and transparent manner. Operational research projects in libraries, national archiving, journals, local and international ^1, 4^ scholarly institutions and centers are partially or yet to be fully tapped into maximizing and ensuring improvements of health and disease information, knowledge and empowerment for all generations ^5^.

Previous literature reviews have shown that open access data and information are of great importance and valuable assets in information sharing, education exchanges and capacity development. This FOA necessity has practically been laid bare by the recent from West Africa Ebola to Zika epidemics crisis where experts across fields including clinical' neonatal and pediatrics have been challenged. Henceforth, academic journals, libraries sources, local and internal Non-governmental organizations (NGOs) data, data from research funded or non-funded projects, centers and institutions should be committed to FOA data and results sharing relevant to the current Zika public health crisis and future emergencies for rapidly emergency mobilization and response. Moreover, the approach has proved to be useful in translation and application of proven and reliable knowledge in guiding effective decision making policies, lifestyle adaptations and contextual programs and strategies in improving public health social economic development and well-being of local and global community ^2, 4, 6, 7^. Most data and information often used in global policies and initiatives are either guaranteed as free by the World Health Organization (WHO) and partners philanthropic organizations, whereas the bulk of support references and documents are not readily accessible to most African scholars, but mainly to policy-makers and implementers ^8^. Equally, limiting access to younger generations of researchers and students who cannot afford the fee to access publication in high impact placed journals, provide highly condensed information not easily informative to those in much need.

The free open access core concept can be characterized by removal of price barriers, no subscription fees and permission barriers, no copyright and licensing restrictions to royalty free literature, to make data and information available to all populations ^2, 8^. “Fee free open access to health data and information for all generations offers a new public health paradigm shift and opportunities to meet the knowledge, lessons learnt and experiences gaps and needs in Africa. Advocacy and mitigation on lack to limited access of existing and emerging data, and information sharing is necessary in embracing regional and global open access. These novelties in information sharing approaches towards collective learning and participative engagement for sound knowledge and empowerment for better health, information exchange for equity in quality education and utilization are paramount for human and societal benefits. It is of fundamental importance to increase multi-disciplinary and inter-sectoral partnerships and collaborations not only to understand and fill the gaps through joint or independent research, but also to be able to use and mine unrestricted data and information for public health good, economic growth and sustainable development ^9, 10^. Although decades of funded and non-funded programs and projects in both developed and developing countries have generated millions of publications and databases on emerging and infectious diseases of poverty ^2, 4, 6^. The impact of policy-translation of lessons learnt and experiences gained are seldom and limited in applications mostly in developing countries. As most LMICs are still challenged with weak health systems and low literacy mainly in remote rural areas and areas of political instability, inadequacies in health funding allocation and resources capacity, poor accountability and governance are present. Moreover, inefficiencies in management and lack of a multi-sectoral approach to access and use local or national data repositories in a structured manner prevent both mainstream national and regional economic development ^11^. Furthermore, the usefulness in forecasting, prevention and management or smart response of emergency situations and disasters are yet to be fully documented and demonstrated in Africa. FOA viability and benefit in most tropical endemics and epidemics-prone developed and developing countries affirmed that the vast majority of metadata and database platforms are still locked (inaccessible and unavailable) for public use and untapped to global community multi-dimensional gains.

Increasing unrestricted and FOA use or reuse as well as timely reporting or communication capabilities strategies are urgently needed in promoting and scaling up data and information sharing and exchange between African researchers, partners and collaborators ^1, 3, 12, 13^. The strength of scaling FOA in developing countries will entail but not limited to: 1) increasing real time and effective knowledge- or evidence-based translation of proven and validated approaches, 2) strategies and tools in strengthening health systems and revamping early and timely access to much needed information by policy-makers, and 3) enhanced guided health financing and capacity development by health institutions and related stakeholders, and strengthening contextual programs and activities planning, transparency and accountability.

This paper assesses the values and benefits of open, free of charge data and information access and availability in strengthening health systems policies, financing, promoting knowledge-based programs and targeted interventions directed to forecast, prevent, reduce and/or manage the growing emerging threats and epidemics as well as infectious diseases of poverty in LMICs, especially in Africa.

## FOA resources and public health needs and demands in LMICs

The growing burden of emerging epidemics and infectious diseases have been documented in demoting health systems in rural and urban settings in Africa. It is important to assess and understand why and how open data and information access is needed in the context of health and diseases epidemics. Also, what capacity development and training are needed to translate these various valuable datasets and database assets if freely available into knowledge-based innovations needed to revolutionize Africa and global health capabilities, and opportunities to prevent and control emerging epidemics and infectious diseases of poverty ^1, 3, 13, 14^.

The current trends of globalization of trade and travel, intense urbanization, economic slowdown are coupled with rising of double epidemics burden (emerging infectious diseases and chronic diseases). Thus, there is an urgent need for open data and information access promotion, advocacy and awareness. This is critical in strengthening and improving the strategic value and usefulness of knowledge-based innovations, teaching and learning, key sources and assets of policy transformation oriented research and primary care innovations (e.g., routine to universal immunization, essential medicines and nutrition). Adopting and adapting open access proven lessons learnt and experiences to alleviate sufferings and poverty, health literacy access and delivery inequities amongst vulnerable populations in Africa is very important ^2, 5, 15, 16^. The evolving use of electronic data and digital delivery platforms to support open access interactive literacy, communication and empowerment of health is a vital need in increasing care acceptability, uptake and scaling up positive cultural ad behavioral changes relevant for communicable and non-communicable diseases vigilance and resilience ^1, 17, 18^. However, with restricted content access, such anticipated evolution in terms of accurate timing and relevant knowledge among experts remains to be a blatant wish as technology and information are not mutually exclusive. Digital technology is only but a driver of available content and hence it thrives, and finds usefulness in the context of information, particularly transformative evidence for universal global health resources access and sharing benefits for all.

Health financing or national resource allocation requires as much open data and information access, analysis, effective and reliable interpretation for outcomes-based sustainable and equitable early decision health financing and funding in achieving local and regional Universal health coverage (UCH) and sustainable development goals (SDGs). Moreover, this new paradigm has the capacity to strengthen and allow exploration of potential local and national health systems, insurance schemes implementation, uptake and coverage impacts as well as legislative and institutional reforms and regulations to enable community and stakeholders commitments and investments ^13, 19^.

## Implications of strengthening knowledge-based innovations and health systems in Africa

Proactive efforts in promoting radical data and information openness and defining criteria tailored to sharing capabilities and transparency are critical and innovative approaches to create monetary and non-monetary benefits ^13, 16^. Data-driven approaches and strategies provide an immense opportunity to understand, define and generate databases that can be used for predictive primary care and innovations in the short- and long-term in diverse scenarios ^14, 16^. The substantiation is argued by knowledge-based open data or information access platforms and proven models that are urgently needed in promoting useful and efficient public health intelligence, health programming and ample financial allocation in disease prevention and control in line with the Abuja declaration in 2000 and health for all of the Africa Union vision 2063 (AU, 20163) and Pan-Africanism agenda ^13, 14, 17, 20, 21^.

In order to attain and optimize the Pan-Africanism aspiration to FOA to current findings and evidence that help shape our decision making process, it is imperative to consolidate online platforms and resources to one stop shop for evidence in different genres. Since timely access to accurate data and information are essential to improving the quality of knowledge and intervention effectiveness, to information scientists alongside librarians globally. Predominantly, with the challenges of electricity shortage and costly internet services, most open access African users’ tendency is increasingly familiar with Google and other internet search engines to discover or access information. Hence, any FOA platform should be user-friendly, non-bias choice, interoperability and flexible. That is, accessibility should not depend on articles being accessed via a special portal or proxy server or publishing platforms, or via complex authorization systems, but should be readily and freely available to all re-users or users consumers and redistribution within the ethical and legal framework. Information should be readily reached without barriers targeting all cadres of technocrats from those with basic training and skills to the advanced. Tested models have proved this relevance and lesson for progressive improvement can be adopted ^16, 17, 22^. One of the implications of not doing so is not being able to find information easily using online systems ^14, 22, 23^. Consequently, researchers and policy-makers and implementers in LMICs have to spend enough time to be informed, consolidate and synthesize what types of information and knowledge can be adaptable, scalable, cost-effective and translated in intervention and best practices ^14, 22, 23^. As a way out in many occasions, when we develop policies for research and programs delivery, we as institutions or individuals take slightly different routes to find the evidence that helps shape our decision making. We often end up relying on a restricted range of platforms, consortia systems and institutional networks that are only readily available small scale data and database evidence online ^20^. Hence, without objectivity and in absence of credible context relevant platforms, we are prone to use biased and/or misplaced approaches policies particularly in the health systems improvement endeavor ^14, 20, 23^.

## Harnessing FOA and resources sharing advocacy and mitigation platforms and policies

FOA platforms prospects are multiple to African scholars, researchers and their collaborators real time and frontline data and other research outputs contextual determinants and scenarios will preferentially entail consolidating R&Ds that are alternatives to the prevailing publishing proprietary models to support open access to health resources. For instance, prioritization databases combine available genomic, genetics and bioinformatics data for each priority genre with automatically extracted and manually curated information for genetic counseling to personalized medicine. Also, in questioning or responding to further literature and other databases research gaps relevant to clinical and analytical practices, putative drug and vaccine target(s) discovery for threatening chronic diseases. Investing substantial efforts in open database mining also permits prioritization, actionable and customized evidence, potential drug and vaccine targets discovery ^3, 4, 11, 18^. Such harnessing may entail the development of research and innovation portfolios focusing on critical public health gaps where traditional approaches are failing, and leveraging proven evidence and lessons learnt on what works and what does not work. As such, the aim would be to attain a long term health agenda and capacity building mitigation via research approaches, cost-effective, timely and progressive innovations ^24, 25^.

Authors advocate to governments, policy-makers and implementers, researchers, academicians, health professionals and other stakeholders including the community to endorse open access public health resources platforms implementation at all levels. There is also need to develop appropriate mechanisms and strategies to promote open access capacity building and empowerment, enhanced health and disease literacy and education through sharing and exchanges, innovative policies and frameworks with advances in digital technologies, establishment of data and information quality control and assurance principle and guidelines, well-coordinated and coherent metadata and database management for evidence operational research and clinical decision making interventions ^26, 27^. The value and credit of FOA does not only promote health and disease literacy, but offer opportunities for mutual sharing of various educational materials, learning and empowerment on maximizing on the use or reuse of the data mining and managed for short- and long-term public health benefits, global health security and wellbeing.

FOA agenda to health workers, professional and providers and communities offers new opportunities in providing affordable, robust, real time and free user friendly and sustainable datasets and databases ^13, 18, 24, 25^. While proactive efforts in reducing or minimizing the various barriers and challenges of FOA uptake and implementation capacity including intellectual property rights, confidentiality, legislation and data use agreements amongst stakeholders (including the lay communities, institutions of learning and studentships) still persist, the value of open access is real ^13, 16, 18, 24^.

## Open access resources and public health benefits in sub-Saharan Africa

The value of freely accessible and available scarce and/or other profuse data, database and information through FOA for public health systems offers tremendous opportunities to strengthen and fasten emerging threat and epidemics including persistent infectious diseases of poverty modeling in preparedness, prevention and control. Moreover, promoting robust evidence-based health and disease surveillance, response planning and funding underscore the social, ecological and economic burden, and opportunities for governments, stakeholders and vulnerable populations ^13, 19, 20, 22, 26, 27^. Nonetheless, FOA and information sharing potential benefits and gains should include but not limited to:

(1)  Enhancing new public health paradigm and innovations in collective and participative education, timely reporting and increasing dissemination and effective trans-boundary risk communication towards democratization of heath data and information for quality health and wellbeing.
(2)  Accelerating proven acceptability and uptake tactics and sustainable mechanisms such as expanded vaccine(s) immunization or mass drug administration in scaling up the coverage and effectiveness to prevent disability and death; uses of wearable technology and sensors in early detection, tracking and monitoring of vectors and/or pathogens and management of associated diseases including non-communicable diseases mitigation and lifestyle adaptations strategies.
(3)  Upholding continuous open access resources advocacy, education and awareness for all in securing universal health coverage, SDGs and “FOA health information for all generations”.
(4)  Nurturing new commitment and investment in novel proven approaches, methods and tools in strengthening local and regional health systems capacity development (infrastructures and resources) in operational and translation research from diverse resources and sources.
(5)  Promoting the value of free, real-time data and information access and availability to all parties in transforming knowledge-based translation into health policy decisions and guiding health priorities financing and public health actions.
(6)  Improving integration and use of information to support evidence-based integrated public-private health and related sectors partnerships (local private sector, bilateral and multi-international) and community-based programs and projects participative ownership.
(7)  Fostering innovative interventions and best practices amongst professional, health workers and the community resilience and participative engagement in response to emerging threats and disasters.
(8)  Facilitating lifetime interactive learning, increasing knowledge, empowerment and resilience in emerging epidemics and infectious diseases vulnerability surveillance and monitoring measures.
(9)  Promoting ethical, legal and international regulations and by-laws applications in safety and security.
(10)  Promoting local, national and regional “One Health” approach in tackling in integrated manner regional and global epidemics of zoonotic infectious diseases prevention, preparedness, control and elimination agenda integration, uptake and utilization for impact.
