# Supplementary material for: Can free open access resources strengthen knowledge-based emerging public health priorities, policies and programs in Africa?
Source: F1000Res. 2016 May 9;5:853. [Version 1] doi: 10.12688/f1000research.8662.1 (PMC4955019; doi:10.12688/f1000research.8662.1)
Supplement: Peer review history

# Peer review: Referee response for version 1

**DOI:** 10.5256/f1000research.9321.r14730

**Published:** 2016-07-15

**Authors:** Daniel Achinko

**Affiliations:** 1 University of the Witwatersrand, Johannesburg, Johannesburg, South Africa

This article was carefully thought of by the authors in relation to the current disease burden faced by the world and its people. The concept of FOA as a driving force for better networking on the knowledge of disease burden, has been an area of concern by researchers, health organizations and the various government bodies around the world. Research based information generated by different labs using high throughput information has been the driving force for obtaining large funding, reasons why the published information cannot be freely accessed in some journals. The resulting challenge is the lack of implementation of the right approaches to disease outcomes in LMICs due to low comprehension of the disease epidemiology and genetic factors. There is actually need for world governing bodies to sit at their conferences and make FOA a priority so that researchers in LMICs, through documented information in accessed journals, could collaborate with authors who have published widely in related fields of interest, helping them contain the disease epidemic using the right approaches before it spreads. The world now is a global village and should be treated as such, hence institutions at all ends of the globe should be able to create some form of partnerships for training and sharing data for the advancement of better health for all.

 The authors actually focused on their indicated topic and brought out related challenges, which suggests reasons for poor approaches to the current disease burden. Their proposals highlight steps which every player involved in decision making at various levels of the disease burden for better health, need to consider for us to attain the health goals put in place.

 I recommend this article as a document for all involved in the research and health field.

I have read this submission. I believe that I have an appropriate level of expertise to confirm that it is of an acceptable scientific standard.

# Peer review: Referee response for version 1

**DOI:** 10.5256/f1000research.9321.r14072

**Published:** 2016-05-31

**Authors:** Oyewale Tomori

**Affiliations:** 1 Department of Microbiology, College of Natural Sciences, Redeemer's University, Lagos, Nigeria

The authors have addressed the highly important issue of free open access (FOA) as a means of tackling emerging epidemics and infectious diseases burden in Africa. They argue that unrestricted open access and free use or reuse of relevant and appropriate data together with sharing of data among African researchers, international partners, policy makers and the community will help limit the effect of burden of infectious disease epidemics in Africa. Availability of real time information on virtually all aspects of infections and diseases is required for timely action and response needed to ensure that public health events do not escalate to international concerns with grave consequences.  It must be however observed that unrestricted free open access to data may lead to users being overwhelmed and unable to make sense out of the data, unless there is available capacity and capability for appropriately analysing and synthesizing the data into valuable information  for policy-makers, implementers, care providers, researchers, health-related institutions and stakeholders including the community. This requires increasing and improving human resource capacity side by side with the call for unrestricted free open access. I find the review stimulating and challenging, with title and abstract matching the contents and conclusions which focuses on the benefits of FOA. As they call for FOA, they also call for the need for unrestricted but planned collaboration between all stakeholders under the “One Health” umbrella.

I have read this submission. I believe that I have an appropriate level of expertise to confirm that it is of an acceptable scientific standard.
